# Back pain in an adolescent: not just a sore spine!

**DOI:** 10.3332/ecancer.2026.2062

**Published:** 2026-01-20

**Authors:** Bothello Mark Richard, Shilpa S Cholachagudda, Pankhudi Priya, Sidharth Totadri, Ranjini Srinivasan

**Affiliations:** 1 Department of Paediatrics, St. John’s Medical College and Hospital, Bengaluru 560034, India; 2 Department of Pediatric Hematology Oncology and Bone Marrow Transplant, St. John’s Medical College and Hospital, Bengaluru 560034, India; a https://orcid.org/0009-0006-8016-9501; b https://orcid.org/0009-0008-1198-3248; c https://orcid.org/0009-0006-7577-3907; d https://orcid.org/0000-0001-8092-7882

**Keywords:** adolescent, arthritis, leukaemia, spine

## Abstract

Acute lymphoblastic leukaemia (ALL) can mimic diverse musculoskeletal conditions, often resulting in diagnostic delays. Genetic predisposition to various cancer syndromes further complicates the clinical picture, influencing disease presentation and treatment response. We report an adolescent boy who presented with a 2-month history of episodic fever, persistent low back pain and non-migratory joint pain, with a history of growth failure, developmental delay and seizures since childhood. There was a history of malignancy in multiple family members. The clinical examination revealed no features suggestive of systemic involvement. The joint examination revealed swelling around the knee joints. Initial work-up for chronic infections and autoimmune diseases was negative. Magnetic resonance imaging spine findings of multiple T2 hyperintense lesions warranted a bone marrow examination, which confirmed the diagnosis of Ph+ B− ALL. Molecular analysis revealed a pathogenic heterozygous missense variant in the TP53 gene, leading to the diagnosis of Li-Fraumeni syndrome. This case highlights the importance of recognizing musculoskeletal symptoms as a potential presentation of ALL. Early consideration of leukaemia in the differential diagnosis can prevent delays in treatment, ultimately improving outcomes.

## Introduction

Low back pain in children is a non-specific symptom but a fairly common problem with a lifetime prevalence ranging from 9% to 69% and thereby increasing with age [1]. The aetiology of low back pain varies from mechanical and inflammatory causes to infectious and more sinister neoplastic processes. Initial laboratory investigations, including blood counts, a peripheral smear and inflammatory markers, often give clues towards a probable aetiology and imaging may be used to provide additional insight into the diagnosis whenever indicated.

Although musculoskeletal (MSK) manifestations are well-documented in 40%–60% of acute lymphoblastic leukaemia (ALL) cases, the presence of arthritis or arthralgia without organomegaly and with a normal haematological profile can often result in misdiagnosis . A retrospective analysis of 63 children with MSK presentation who underwent bone marrow examination reported findings suggestive of leukaemia in 30.15% patients [2]. Misdiagnosis of a neoplastic process not only causes treatment delays but also results in unwarranted use of systemic steroids . This case report highlights the importance of maintaining a high index of suspicion for atypical presentations of leukaemia, demonstrating that low back pain in an adolescent can be an ominous symptom of an underlying malignancy. Although paediatric cancers arise *de-novo* in most cases, a small proportion can have underlying cancer predisposition syndromes. The current case underscores the importance of family history in identifying such syndromes.

## Case report

A 17-year-old adolescent male, presented with persistent low back pain, of 2 months duration . The pain was insidious in onset, dull aching and persistent throughout the day with minimal relief with oral analgesics. The pain was more in the sitting posture, with the patient reporting difficulty in bending forwards . This was accompanied by non-migratory, non-erosive , additive joint pain predominantly involving the knee joints, which was more pronounced at night and did not respond to conventional anti-inflammatory medications. The pain was not associated with stiffness but was severe enough to disrupt his routine daily activities. There was a history of intermittent, episodic low-grade fever since the onset of back pain, which responded to antipyretics . The patient also had accompanying malaise and anorexia with significant weight loss.

He was born at term to third-degree consanguineous parents with a normal birth weight. The antenatal and postnatal periods were uneventful. There was history of delayed attainment of developmental milestones and epilepsy for which he was on antiseizure medications . He was completely immunised, and his scholastic performance was average, with poor social and communication skills.

**Figure 1.**
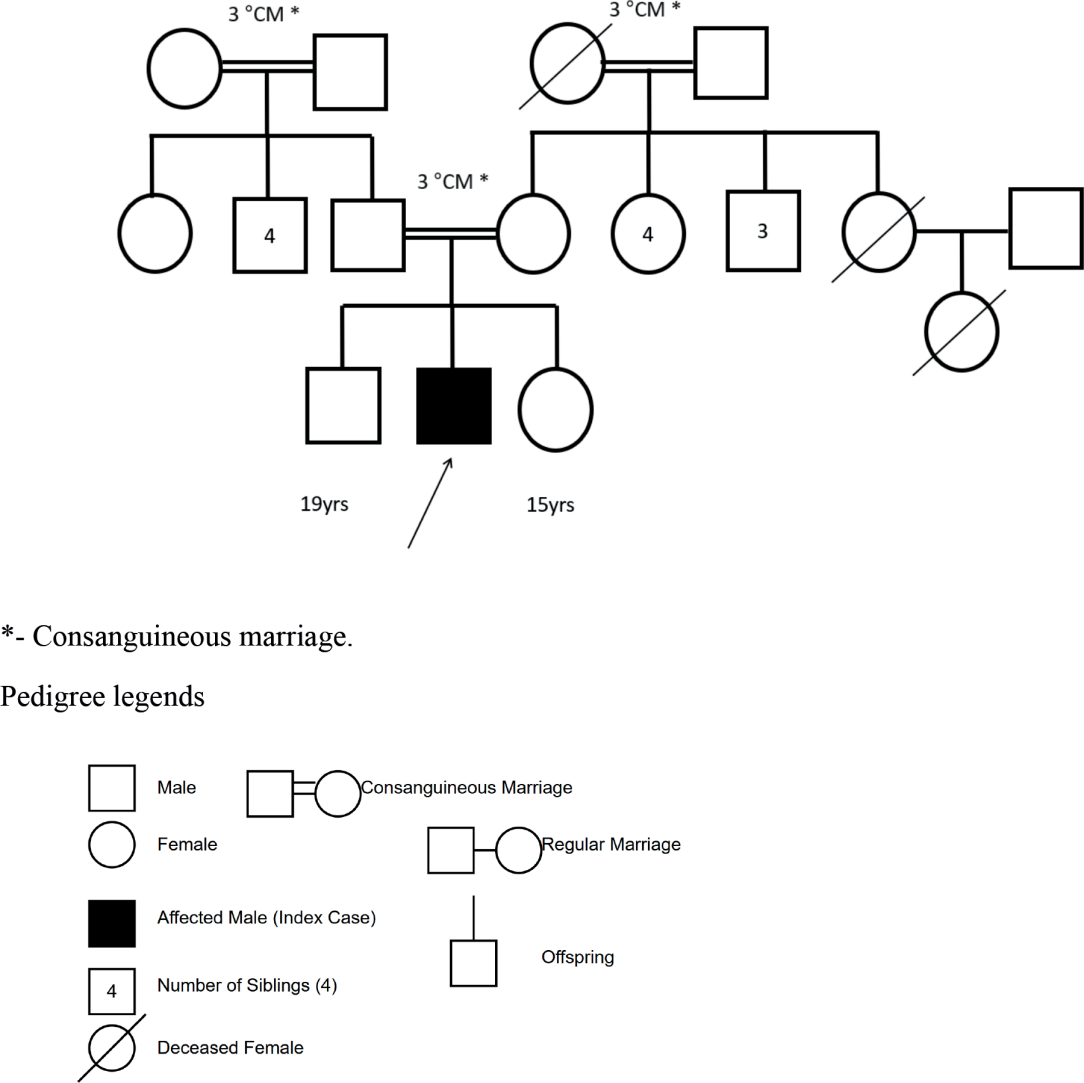
Three-generation family tree of the index patient (indicated by arrow).

There was no history suggestive of tuberculosis among household contacts or autoimmune disease among first-degree relatives. A positive family history noted was the presence of breast cancer in the patient’s mother and uterine cancer in the maternal grandmother. The first cousin was recently diagnosed with acute leukaemia a few months prior (Figure 1).

At presentation, he was pale and emaciated. He appeared dysmorphic with triangular facies, small-sized head, prominent ears and hypertelorism . His vital parameters were within normal limits. Anthropometry revealed severe thinness , stunting and microcephaly. There was a café au lait macule measuring 3 × 1 cm over the abdomen . There was no icterus, pedal oedema or significant lymphadenopathy . There were no skin bleeds. Oral cavity and dentition appeared normal. There were no limb anomalies and spine examination were normal.

MSK system examination revealed a normal gait and posture. He was unable to bend forward and touch his toes and spine movements were restricted only in forward flexion. Joint examination was normal with no features of inflammation. There was no joint line tenderness or limb length discrepancy. Systemic examination revealed no organomegaly. Neurological examination was normal and there were no focal deficits. Differentials considered at this point included chronic infections such as tuberculosis and retroviral infection affecting bone and joints, inflammatory conditions such as ankylosing spondylitis, juvenile rheumatoid arthritis and systemic lupus erythematosus and neoplastic conditions considering the strong family history.

**Figure 2.**
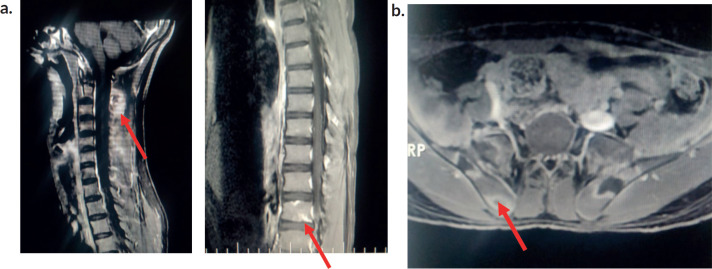
Magnetic resonance imaging of the spine . (a): MRI spine-post contrast enhancement noted in C2 and C6 vertebral bodies.Osteolytic lesion noted at C2. Ill-defined enhancement noted in the paraspinal muscles posterior to C2 and thoracic vertebra. (b): MRI Hip -Ill-defined STIR hyperintense foci noted in bilateral iliac bones and sacrum (ala) with peripheral enhancement.

Initial investigations (Table 1) showed moderate anaemia, with normal total leucocyte counts, normal platelet counts and elevated inflammatory markers. His peripheral smear was normal with no atypical cells. The chest radiograph was normal. Ultrasonogram (USG) of the abdomen revealed mild splenomegaly and knee joint USG showed no evidence of synovitis or effusion. Work up for rheumatological conditions (Table 1) was negative. Due to persistent lowback pain and elevated inflammatory parameters, Magnetic resonance imaging (MRI) of the spine and pelvis was performed, which showed ill-defined T2 hyperintense lesions involving multiple cervical and thoracic vertebrae with Short Tau Inversion Recovery (STIR) hyperintense lesions in bilateral iliac bones and sacrum. There was an osteolytic lesion noticed in the spinous process of C2 with periosteal reaction . There was no leptomeningeal enhancement (Figure 2a and b).

**Figure 3.**
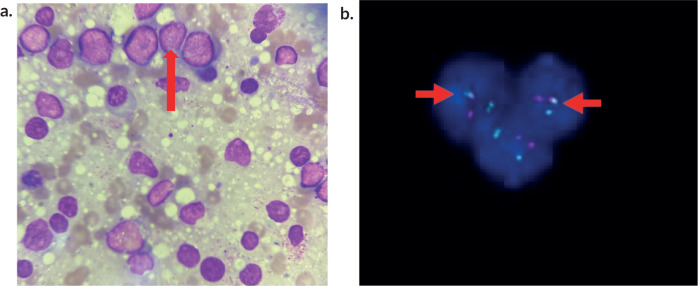
(a): Bone marrow smear cytology. Bone Marrow smear revealing prominent lymphoblasts (arrow) and myeloid cells in various stages of maturation. (b): depicting FISH analysis. BCR/ABL1 gene with fusion positivity in 15 % of cells (in interphase).

Suspecting a possible neoplastic aetiology, bone marrow aspiration and biopsy were done which confirmed acute leukaemia (Figure 3a).

Extended flow cytometry was positive for B-cell precursor markers (CD34, TdT, HLA) and B cell markers (CD 19, 20, 10). Fluorescent *in situ* hybridisation (FISH ) showed interphase cells and BCR/ABL-1 gene positivity in 15% of cells (Figure 3b).

Based on the above findings, the patient was classified as high-risk B cell ALL and treated as per Indian Childhood Collaborative Leukaemia 2014 regime . He is currently on the maintenance phase of chemotherapy [3]. He was also started on Imatinib Mesylate due to his Philadelphia chromosome positivity. Assessment of minimal residual disease activity at the end of induction was negative. A clinical exome sequencing performed in the child after achieving remission with chemotherapy revealed a pathogenic heterozygous missense variant in exon 8 of the TP53 gene resulting in amino acid substitution of leucine for valine at codon 272, leading to the diagnosis of Li-Fraumeni syndrome (LFS), a rare cancer predisposition syndrome. On subsequent follow-up 9 months later, there has been a significant and sustained improvement in the clinical profile.

## Discussion

MSK involvement in childhood leukaemia occurs with a frequency ranging from 7.1% to 62.3% [4]. Malignancies causing bone pain in children include leukaemia, lymphoma, neuroblastoma and sarcoma. Postulated mechanisms for articular symptoms include joint infiltration, synovial reaction, periosteal or capsular lesions, intra-articular or peri-articular haemorrhage, neoplastic cytokine secretion or infection [5]. About one third of paediatric patients with ALL initially present with MSK symptoms [6]. These may include limb pain, bone pain, joint pain and gait abnormalities such as limping, as described in a systematic review where limb pain constituted 43% of all pooled symptom frequencies [6]. Vertebral body collapse and back pain in leukaemia in the absence of overt systemic symptoms and signs are not well described in the literature . Leukaemic arthritis may be misdiagnosed as juvenile idiopathic arthritis and steroid initiation in these patients may further contribute to diagnostic delay. Severe pain disproportionate to physical findings, pain in an atypical location (metaphyseal region), nocturnal pain, poor response to anti-inflammatory drugs and development of early osteopenia or lytic lesions are clues in favour of a possible neoplastic process [7]. The severe nocturnal pain, joint findings disproportionate to pain severity and poor response to pain killers were pointers towards a non-rheumatological pathology in this patient .

A retrospective review describing the clinical profile and outcomes of children with MSK presentation of leukaemia (*n* = 81) observed that rheumatological involvement was more likely in children with ALL (88.9%) as compared to those with acute myeloid leukaemia (11.1%), notably B cell ALL (69.4%). The study also reported that children with MSK involvement were more likely to have fever. However, skin bleeds and splenomegaly were found to occur more frequently in the group with MSK presentation (*p* < 0.001 and 0.043, respectively ), which was not seen in our patient. Those with MSK presentations had higher haemoglobin and platelet counts with lower WBC count as compared to those without MSK involvement [4]. The absence of peripheral blasts was significantly higher in the group with MSK manifestations (17.3% versus 9.6%, p = 0.04). These laboratory findings corroborate with what was observed in our patient as well.

MRI has become a useful tool to diagnose and stage haematological malignancies and is preferred over other modalities of imaging to evaluate bone pathology . The pattern of marrow signal (i.e., diffuse, variegated or localised) may indicate the underlying disease process. A diffuse involvement throughout the marrow is usually observed in acute leukaemia with a decrease in signal intensity on T1 weighted images. T2 or STIR images help in detecting the abnormal increased marrow signal intensity with fat-suppressed T2W signals relative to the intervertebral discs and paravertebral muscles [8, 9]. Other bony abnormalities detected by imaging modalities in children with haematolymphoid malignancies include lucent metaphyseal bands, smooth to irregular patterns of periosteal reaction, pathological fractures, osteopenia, geographic lytic lesions and sclerosis [10].

Yoshikawa *et al* [11] in their case series demonstrated the usefulness of MRI in children with leukaemia, especially in situations where MSK manifestations appear prior to haematological abnormalities in the peripheral blood. In this series, the lag period between the onset of MSK symptoms and the diagnosis of ALL varied from 20 days to as long as 6 months. During this period, the patients were misdiagnosed with pyogenic osteomyelitis or arthritis due to high signal intensity on T2 - weighted imaging of the involved bones or joints. The authors concluded that diffuse low signal intensity on T1 - weighted images, especially in regions adjacent to localised sites of MSK symptoms, although not specific, when considered in combination with high signal intensity on T2 weighted images , may provide valuable information to diagnose insidious leukaemia. Similar neuroimaging findings observed in this patient prompted us to proceed with further bone marrow studies [11].

The family history of malignancy prompted the investigators to search for a possible underlying hereditary cancer syndrome. The index case fulfilled the Chompret criteria for LFS [12]. LFS is an autosomal dominant hereditary condition caused by a germline mutation in the tumour protein 53 (TP53 gene), leading to an increased risk of multiple cancers. The five core malignancies described in this condition are adrenocortical carcinoma, breast carcinoma, central nervous system tumors, osteosarcomas and soft-tissue sarcomas [13]. Leukaemias compromise a small percentage among the malignancies in LFS, with maximum predisposition among children less than 15 years of age [14, 15]. The most common leukaemia is hypodiploid ALL, followed by B-cell ALL [15]. Periodic surveillance , including clinical workup and regular imaging on a quarterly basis , is recommended in these patients [16, 17].

The literature review did not show any association between LFS and developmental delay . Kuhlen *et al* [18] described that some of the oncogenes and tumour suppressor genes causing cancer susceptibility syndromes overlap with those involved in autism , which needs further studies to unveil the association between developmental delay and LFS. Though there is no cure for LFS, regular surveillance can facilitate early diagnosis of cancers. Individual cancers are treated as per standard protocols. However, radiation therapy should be used cautiously due to the increased risk of radiation-induced cancer [15]. This patient has been on regular follow up and is being monitored periodically for early detection of new cancers.

## Conclusion

Acute leukaemia is known to mimic MSK disorders. Pain disproportionate to physical examination findings, unexplained systemic symptoms, low normal platelet count and algorithmic approach to changes in signal intensities in MRI spine were the clues in this patient that prompted the search for an alternate diagnosis. A detailed family history is indispensable to the suspicion of underlying hereditary cancer predisposition syndromes in children with cancer.

## Abbreviations

ABL: Abelson tyrosine kinase gene at chromosome 9, ALL: Acute lymphoblastic leukaemia, ALP: Alkaline phosphatase, BCR: Break point cluster gene at chromosome 22, CD: Cluster of Differentiation, CHPS: Childhood predisposing syndromes, GGT: Gamma glutamyl transpeptidase, HLA: Human Leukocyte Antigen, LFS: Li Fraumeni Syndrome, MRI: Magnetic resonance imaging, MSK: Musculoskeletal, N:C: Nuclear to cytoplasmic ratio, STIR: Short tau inversion recovery, TdT: Terminal deoxynucleotidyl transferase, USG: Ultrasonography.

## Conflicts of interest

All authors have declared no competing interests.

## Funding

No funding was obtained for this case report.

## Author contributions

MB, SC, PP and RS: wrote the manuscript. RS, ST, SC and PP: Managed the patient in the hospital and have followed up the patient. All authors have read and approved the final manuscript.

## Ethical approval

The Institutional Ethics Committee (IEC) of St. John’s Medical College has approved this case report. IEC Study reference number is 315/2024.

## Figures and Tables

**Table 1.** Investigations of the patient in the hospital.

Investigation	Patient value	Normal range
Haemoglobin	9.3 g/dL	11.5–15.5 g/dL
Total leucocyte count	6,280 cells/µL	4,000–11,000 cells/µL
Differential count	Neutrophil 72 % Lymphocyte 15% Eosinophil 0% Monocyte 8 %	
Platelet count	162,000 cells/µL	150,000–400,000 cells/µL
Packed cell volume	28.6%	40%–54%
Erythrocyte sedimentation rate	119 mm/h	0–9 mm/h
Peripheral smear	Red blood cells – are normocytic normochromic with few microcytes, ovalocytes, tear drop cells and polychromatophils Leukocytes – are normal in total number with few reactive lymphocytes Platelets – adequate, no atypical cells Normocytic normochromic picture	
Creatinine	0.68 mg/dL	0.69–1.1 mg/dL
ALP	721 U/L	56–167 U/L
GGT	385 U/L	5–55 U/L
C-reactive protein	>32 mg/dL	0.02–0.5 mg/dL
Procalcitonin	0.13 ng/mL	<0.5 ng/mL
Calcium	9.2 mg/dL	9.2–10.5mg/dL
Phosphorous	4.1 mg/dL	2.3–4.7 mg/dL
B12	>256 pmol/L	25–165 pmol/L
Vitamin D	17.6 ng/mL	Deficient <20 ng/mL
CPK	12 U/L	30–200 U/L
Uric Acid	3.2 mg/dL	2.5–6.2 mg/dL
Work up for tuberculosis: Gastric aspirate for acid fast bacilli (AFB) and gene expert Bone marrow for mycobacterial culture	Negative	
Human Immunodeficiency virus	Non-Reactive	
Rheumatoid factor	<10 µIU/mL	Up to 18 IU/mL
C3	119.78 mg/dL	90–180 mg/dL
C4	38.87 mg/dL	9–36 mg/dL
ANA By Immunofluorescence	Negative	
DCT	Negative	
HLAB27	Negative	
Bone marrow aspirate	Bone marrow aspirate showed hypercellular marrow with 31 % Erythroid precursors and 22% lymphocytes with 25% medium to large blasts with high N:C ratio, coarse chromatin 1–2 nucleoli & scant cytoplasm. Features suggestive of Acute leukaemia	

ALP: Alkaline phosphatase, GGT: Gamma glutamyl transpeptidase
